# Increased pain unpleasantness and pain-related fMRI activation in the periaqueductal gray in Alzheimer's disease

**DOI:** 10.3389/fpain.2022.914473

**Published:** 2022-10-28

**Authors:** Alison R. Anderson, Todd B. Monroe, Mary S. Dietrich, Stephen P. Bruehl, W. Larkin Iversen, Ronald L. Cowan, Michelle D. Failla

**Affiliations:** ^1^ Center for Complex Care, Self-Management and Healthy Aging, The Ohio State University, College of Nursing, Columbus, OH, United States; ^2^ School of Nursing, Vanderbilt University, Nashville, TN, United States; ^3^ Institute of Imaging Science, Vanderbilt University Medical Center, Nashville, TN, United States; ^4^ Department of Biostatistics, Vanderbilt University Medical Center, Nashville, TN, United States; ^5^ Anesthesiology, Vanderbilt University Medical Center, Nashville, TN, United States; ^6^ Departments of Psychiatry and Anatomy and Neurobiology, University of Tennessee Health Science Center, College of Medicine, Memphis, TN, United States; ^7^ Department of Psychiatry and Behavioral Sciences, Vanderbilt University Medical Center, Nashville, TN, United States

**Keywords:** Alzheimer disease, dementia, pain, periaqueductal gray, neuroimaging, fMRI, BOLD, psychophysics

## Abstract

**Background:**

Pain continues to be underrecognized and undertreated in people with Alzheimer's disease (AD). The periaqueductal gray (PAG) is essential to pain processing and modulation yet is damaged by AD. While evidence exists of altered neural processing of pain in AD, there has not been a focused investigation of the PAG during pain in people with AD.

**Purpose:**

To investigate the role of the PAG in sensory and affective pain processing for people living with AD.

**Methods:**

Participants from a larger study completed pain psychophysics assessments and then a perceptually-matched heat pain task (warmth, mild, and moderate pain) during a functional MRI scan. In this cross-sectional study, we examined blood oxygenation level-dependent (BOLD) responses in the PAG and other pain-related regions in participants with AD (*n* = 18) and cognitively intact older adults (age- and sex-matched, *n* = 18). Associations of BOLD percent signal change and psychophysics were also examined.

**Results:**

There were significant main effects of AD status on the temperature needed to reach each perception of warmth or pain, where people with AD reached higher temperatures. Furthermore, participants with AD rated mild and moderate pain as more unpleasant than controls. PAG BOLD activation was greater in AD relative to controls during warmth and mild pain percepts. No significant differences were found for moderate pain or in other regions of interest. Greater PAG activation during mild pain was associated with higher affective/unpleasantness ratings of mild pain in participants with AD but not in controls.

**Conclusion:**

Results suggest a role for the PAG in altered pain responses in people with AD. The PAG is the primary source of endogenous opioid pain inhibition in the neuroaxis, thus, altered PAG function in AD suggests possible changes in descending pain inhibitory circuits. People with AD may have a greater risk of suffering from pain compared to cognitively intact older adults.

## Introduction

Research demonstrates that pain processing is altered in Alzheimer's disease (AD) (1–6) but the neural basis is not well understood, which may be a reason pain continues to be underrecognized, underreported, and undertreated in this condition (7–12). Unfortunately, 45.8%–75% of people with AD and related dementias suffer from acute and chronic pain regularly (13–16). This represents substantial numbers because there are 6.5 million people living with AD in the United States (US) (17) and over 55 million with dementia globally (18). Preliminary neuroimaging studies indicate that major brain regions involved in pain processing continue to demonstrate activation in response to painful stimuli in AD (1–3), but it remains unknown how this activation contributes to the pain experience in AD. Numerous neuropathological changes that occur during AD (1, 2, 4, 19–22) likely contribute to this altered pain experience (1–4).

Pain is mediated by multiple brain regions working in concert (23, 24). However, for research purposes, pain processing is often described as occurring within separate medial/sensory and lateral/affective pain networks, as well as the more recently posited rostral (pain behavior) pain network (4), in addition to cognitive modulation of pain by the prefrontal cortex (25). Comprised of regions within each of these networks is the descending pain modulatory system (DPMS), of which the periaqueductal gray (PAG) is an essential component (26–29). The PAG contains high levels of opioid receptors and is the primary source of endogenous opioids within the central nervous system (26, 30). The PAG is activated by painful stimulation and the resulting release of endogenous opioids can modulate nociceptive transmission to and from higher brain structures (23, 30, 31). Enkephalins, dynorphin, and beta-endorphin are all endogenous opioids found within the PAG (30, 32). Enkephalins are the most abundant opioid in the PAG (32), and neurons in the PAG manufacture enkephalins (33, 34) and dynorphins (34). Experimental electrical stimulation of the PAG typically inhibits nociceptive signals (27, 35), likely *via* endogenous opioid release (30, 36), which has been shown in rodent models (35) and in humans (37). Opioid receptors in the PAG are also significantly involved in the pain-relieving effects of exogenous opioid analgesics (36, 38–40).

In patients with AD, there is volume loss in the PAG (41) resulting from damage related to amyloid-*β*, abnormally phosphorylated tau, and neurofibrillary tangles that are not found in controls (19–22). These pathological changes could mean that people with AD may have an impaired ability to generate a sufficient endogenous opioid analgesic response to pain or may experience an altered response to exogenous opioid analgesics. A consequence of this potential impairment may be an amplified pain experience in people with AD compared to cognitively intact older adults.

Prior work has provided critical knowledge about the pain experience in mild to severe AD (including Mini-Mental State Exams as low as 8–12; where scores below 24 may indicate dementia) (5), but the literature remains mixed with no consensus of findings. Evidence from psychophysical studies demonstrate that people with AD detect pain stimuli at similar (42–47) or greater intensities (e.g., higher temperatures) (1–3, 48, 49) compared to controls (5). These studies and others have shown that people with AD have pain thresholds (42–44, 47, 48), tolerance (42–44), habituation (44, 47), and psychophysical performance and reproducibility (43) that are similar to those of controls (5). However, several studies have reported that pain unpleasantness, a characteristic specifically related to the affective component of pain, is the same (1–3, 42, 45, 46, 49–51) or higher in AD patients than in controls (1, 5, 6, 44, 47, 51–54).

Because of its high temporal resolution relative to other imaging modalities (55), blood oxygenation level-dependent (BOLD) functional Magnetic Resonance Imaging (fMRI) is a powerful but noninvasive way (56) to investigate neurobiological mechanisms in the brain that influence responses to pain (24, 57–59). Thus far, three published fMRI studies have investigated pain processing and self-reported acute pain in mild to moderate AD (1–3), and a fourth fMRI study investigated resting-state connectivity and, separately, acute observational pain in mild to severe AD (6). Of these four studies, only one investigated fMRI in response to a pain stimulus task (1), while the other three were resting-state studies (2, 3, 6). Although the PAG was implicated in one of the functional connectivity studies of pain responses in AD (2), the only other studies that focus on the PAG in AD are post-mortem histological investigations that demonstrate damage to the PAG during the disease process of AD (19–22). Numerous other studies have contributed additional knowledge about pain in AD, but many are observational in design, focus on self-report or behavioral pain expressions, and/or use only experimental pain psychophysics. In general, few previous experimental studies have investigated how neurobiological changes in AD affect pain processing (3, 4).

In cognitively intact participants, greater pain intensity is generally associated with greater PAG activation (60, 61), likely reflecting the nociceptive stimulus-dependent nature of PAG activation. Little is known about such associations in individuals with AD. Compared to cognitively healthy controls, greater brain activation is found during painful stimulation in multiple brain regions in people with AD (1, 4). In the only previous fMRI task study, Cole et al. found in 14 participants with AD and 15 age-matched controls that activation following painful stimulation was preserved in major brain regions in the pain network and not diminished in individuals with AD (1). Rather, greater BOLD responses indicative of activation during pain were found in cortical regions, thalamus, caudate, putamen, globus pallidus, and cerebellum, alongside reports of greater pain unpleasantness in participants with AD (1).

The purpose of this between-groups cross-sectional study was to test for differences in PAG activation in response to a standardized evoked heat pain stimulus in participants with AD compared to healthy age- and sex-matched controls. We hypothesized increased BOLD responses (activations) in the PAG during heat-induced pain in participants with AD compared to controls because of previous work finding increased activations during pain (1, 4) and previous findings of elevated pain responsiveness in persons with AD (1, 5, 6, 44, 47, 51–54). We also hypothesized that the extent and direction of correlations of PAG BOLD responses with psychophysical responses (percept temperatures and reported pain unpleasantness) would differ by group (62). We predicted there would be greater PAG activation with higher temperatures and higher levels of pain in people with AD compared to controls.

## Materials and methods

### Sample and collection methods

This cross-sectional study included between-groups analysis of fMRI and psychophysical data obtained during an experimental evoked heat pain protocol using data collected previously as part of a larger parent study from which resting-state connectivity (3) and psychophysical (49) results in AD, and fMRI task data of cognitively normal participants (63) have previously been reported. The protocol was approved by the Institutional Review Board of Vanderbilt University and ethical guidelines on human experimentation were followed in accordance with the Declaration of Helsinki.

Participants were originally recruited from geriatric practices at Vanderbilt University Medical Center (49, 63). Inclusion and exclusion criteria from the original data capture during the parent study are described elsewhere (63). In brief, the participants in the AD group had a diagnosis of AD with other causes ruled out, a Mini-Mental State Examination (MMSE) of 10–26, were otherwise relatively healthy, and had a caregiver. Controls were relatively healthy, had an MMSE > 26, and both participants with AD and controls had no pain conditions or analgesic use, and had no contradictions for 3 Tesla (3 T) MRI scanning. To determine the capacity for consent, the University of California San Diego Brief Assessment of Capacity to Consent (64) was used. If needed, participants were able to sign an assent document and surrogate consent was obtained from a caregiver or legal guardian. Participants underwent psychosocial assessments during the larger study (3, 49, 63). These included the Hollingshead Four-Factor SES (65), MMSE (66), Brief Pain Inventory (BPI) (67), Geriatric Depression Scale (GDS) (68), State-Trait Anxiety Inventory (STAI) (69), and MRI safety clearance (49, 63).

To be included in this final analysis, participants had the following: (1) age- and sex-matched controls available, (2) specific MMSE scores for controls of >29 and AD <23, and (3) had fMRI data obtained during the evoked heat pain task protocol. For this analysis, there were 40 participants available with full fMRI and psychophysical data from the parent study (3, 49, 63). We narrowed the MMSE range for this analysis to decrease the chances of including participants with mild cognitive impairment. After sex-matching and removing participants with greater than a 3-year age difference for age-matching, we were left with 36 participants (18 AD, 18 control), comprised of 50% female participants within each group.

#### Psychophysics

The protocol used for the psychophysical methods was modeled after the protocol used by Cole et al., (1, 49, 63) and are detailed fully elsewhere (3, 49, 63). The Medoc Pathway Pain and Sensory Evaluation System is an FDA-approved thermal stimulator that was used to deliver heat pain stimuli (3, 49, 63, 70). The pain psychophysics protocol was conducted outside of the MRI scanner prior to the imaging protocol below. The stimuli began with a baseline of 30°C with an upward ramp rate of 1 °C per second using a 30 mm ×  30 mm thermode positioned on the thenar eminence of the right hand (3, 49, 63). During the ascending heat stimuli, the participants were asked to notify the research assistant when they perceived sensations of “warmth,” “mild pain,” and “moderate pain” (with temperatures at each percept reflecting the percept intensity data). The participants were given instructions by the research assistant of: “I will tell you when the metal cube that is attached to your hand will start heating up, then I will ask you to stop the heat when you feel ‘warmth,’ ‘mild pain,’ or ‘moderate pain.’ I will not ask you to rate any pain greater than ‘moderate pain.’ An example of ‘mild pain’ might be the temperature of a hot bath and an example of ‘moderate pain’ might be the temperature of a fresh hot cup of coffee.” The Vanderbilt University Institutional Review Board required the maximum pain level of the protocol to be the participant's subjective report of moderate pain. Therefore, perceptually-matched pain stimuli, rather than fixed temperature stimuli for all participants, were required. To assess the affective component of pain, participants were also asked to rate the sensation at each percept above on a 0–20 unpleasantness scale (0 = neutral, 20 = very intolerable) (3, 49, 63). To verify that the participants understood, the participant would describe their understanding of the instructions to the research assistant at each step of the study and we further checked that each percept level increased in temperature (e.g., temperatures for mild pain were higher than warmth).

#### fMRI acquisition and preprocessing

Brain images were acquired on a Philips 3T Achieva MRI scanner (Philips Healthcare Inc., Best, Netherlands). During scanning, four runs were administered. Each run included six thermal stimulus blocks of two trials each of the three pain percepts (warmth, mild pain, moderate pain) delivered to the participant in pseudorandomized order to prevent order effects. Each percept intensity was determined during the previously conducted pain psychophysics protocol. Each stimulus presentation had an 8 °C per second-ramp rate and 16-second stimulus duration, with a 24-second rest between stimuli. Parameters were determined by an MRI physicist based on the goals of the study and included both structural and functional runs (63). A standard whole-brain 3-D anatomical T1-weighted/time of flight echo (TFE with SENSE coil) scan was acquired. In each 264-s-duration functional run, 28 field echo planar imaging (EPI) (162 dynamics, 4.50-mm slice thickness with 0.45-mm gap, 2-s time to repeat (TR), 35-ms echo time (TE), 79° flip angle, field of view (FOV) = 240, matrix = 128 × 128 (with a voxel size of 2 mm × 2 mm × 2 mm) scans were acquired (63).

PAG BOLD activation and psychophysical responses during experimental pain were compared between participants with AD and healthy age- and sex-matched controls. While the PAG can independently modulate pain (71), we also explored responses in the larger neurologic pain signature (NPS) (24). This included the regions of interest (ROI) of the insula, thalamus, anterior cingulate cortex (ACC), and secondary somatosensory cortex (S2) derived from the NPS. These brain regions are included in the medial, lateral, and rostral pain networks (4). The fMRI-derived NPS is specific to physical pain and is based on repeated and reproducible BOLD signal activations during an evoked heat pain stimulus (24) and its accuracy has been replicated across multiple studies (72–76). Heat is one of the most common and most reliable (43) evoked pain stimuli used in psychophysical studies of AD and pain.

fMRI pre-processing and analyses were performed using standard methods in Statistical Parametric Mapping (SPM) (77, 78), version 12. Slice timing and motion correction (using standard rigid body registration of intra-scan volumes) were applied to the fMRI data using standard SPM12 techniques. Volumes were co-registered to structural T1-weighted volumes using the first image volume from each fMRI imaging run. Whole-brain images were spatially smoothed with an 8-mm full-width half-maximum Gaussian kernel. Structural data were registered to Montreal Neuroimaging Institute (MNI) space and the resulting transformation matrix was applied to the fMRI data (63). Band-pass filtering of 0.01 to 0.1 Hz was applied. The Robust Weighted Least Squares (rWLS) toolbox (79) was used to correct for motion in the temporal sequence, by deweighting motion outliers. Each subject's average white matter, cerebrospinal fluid, and average motion parameters (in mm) were included in the first level model as variables of no interest.

The PAG ROI was generated from a consensus centroid derived from Neurosynth (80) with MNI coordinates 2, −26, −10, and a 6 mm sphere was formed around this centroid using the MarsBaR toolbox for SPM12 (81). SPM's default small volume correction (SVC) does not perform accurately for very small ROIs (55), such as the PAG. Thus, we adapted Kong, Linnman, and colleagues' methodology (82, 83) using a 6 mm PAG sphere with a threshold level of 10 contiguous voxels with voxel-wise *p* = 0.05 (82). Secondary ROIs (insula, thalamus, ACC, and S2) were generated with the Wake Forest University (WFU) PickAtlas (84) with bilateral masks generated separately (e.g., “right thalamus” and “left thalamus”). This resulted in eight additional ROIs, with nine total ROIs examined including the PAG. For percent signal change, the average signal during pain stimuli at each percept for each ROI was extracted. For the secondary ROIs (insula, thalamus, ACC, and S2), we used the standard SPM 12 FWE correction *p* = 0.05.

### Data analysis

Descriptive statistics were used to summarize the characteristics of the participants with AD and the matched control participants. Due to skewness of the distributions of several of the continuous variables, median and inter-quartile ranges were used to summarize those values; frequency distributions were used for nominal and ordinal categories. Mann-Whitney (continuous) and Chi-square tests of independence (categorical) were used to compare the characteristics of the two groups.

Median and inter-quartile range (IQR) were also used to summarize the stimulus intensity at which each level of pain percept was reported (i.e., stimulus temperature, a reflection of sensory pain) and the perceived unpleasantness of the sensation (affective pain) at the respective percept level. Mixed-effects general linear models were used to test the main and interactive effects of AD status and pain percept level on each of the measures. If interaction effects were statistically significant, pairwise tests within those models were used to compare the stimulus temperature values and affect/unpleasantness responses between the groups at each pain percept level. Skewed data distributions were transformed to normal using either square-root or log as necessary to meet the assumptions of linear modeling methods. Cohen's d effect sizes were generated for each comparison to index magnitude of the observed differences regardless of statistical significance. Correlations of BOLD responses during painful percepts with psychophysical measures obtained outside the scanner were examined using Spearman's rho. Tests of differences between those correlations in the two groups used Fisher's *z-*test of independent correlations. An alpha level of *p* < 0.05 was used for hypothesis testing.

## Results

As shown in Table 1, the majority of the sample was Caucasian and right-handed. As expected, in addition to MMSE score differences, participants in the AD group had higher GDS scores than did those in the control group, as depressive symptoms and MMSE are known to co-vary (3, 85) (*p* < 0.001, see Table 1). Despite differences, all GDS scores were in the non-depressed range, and were therefore unlikely to have impacted study results and were not controlled for during fMRI analysis.

**Table 1:** Demographic and clinical characteristics by study group.

	Total (*N* = 36)	Control (*N* = 18)	AD (*N* = 18)	*** ***
	*N* (%)	*N* (%)	*N* (%)	*p-*value
Education	** **		** **	0.395
≤High school	7 (19.4)	2 (11.1)	5 (27.8)	
Partial college	8 (22.2)	4 (22.2)	4 (22.2)	
College graduate	11 (30.6)	5 (27.8)	6 (33.3)	
Graduate degree	10 (27.8)	7 (38.9)	3 (16.7)	
Handedness				0.070
Right	33 (91.7)	15 (83.3)	18 (100)	
Race/ethnicity				0.546
African-merican	3 (8.3)	1 (5.6)	2 (11.1)	
Caucasian	33 (91.7)	17 (94.4)	16 (88.9)	
	**Median [IQR] Min, Max**	**Median [IQR] Min, Max**	**Median [IQR] Min, Max**	***p-*value**
Age	71.0 [68.0, 78.8] 65, 86	71.0 [67.7, 79.3] 65, 86	72.5 [68.0, 77.5] 65, 86	0.799
MMSE score^a^	26.5 [15.5, 30.0] 10, 30	30.0 [29.0, 30.0] 29, 30	16.0 [11.7, 22.0] 10, 24	<0.001
Average pain^b^	0.5 [0.0, 3.0] 0, 5	1.0 [0.0, 2.3] 0, 4	0.0 [0.0, 3.0] 0, 5	0.709
Pain right now^b^	0.0 [0.0, 0.0] 0, 4	0.0 [0.0, 1.0] 0, 3	0.0 [0.0, 0.0] 0, 4	0.528
GDS-SF score^c^	1.0 [0.0, 3.8] 0, 5	0.0 [0.0, 1.0] 0, 5	3.0 [1.7, 4.0] 0, 5	<0.001
STAI state score^d^	48.5 [45.0, 50.8] 32, 75	50.0 [46.5, 51.5] 32, 75	48.0 [44.7, 50.0] 42, 68	0.293
STAI trait score^d^	47.0 [44.0, 50.0] 32, 56	47.0 [44.0, 50.0] 32, 56	46.0 [42.7, 49.3] 41, 56	0.339

^a^ Folstein Mini Mental State Examination (range = 0–30; 0 = completely cognitively impaired, 30 = completely cognitively intact).

^b^ BPI-SF-Brief Pain Inventory Short Form (range = 0–10; 0 = no pain, 10 = most pain).

^c^ GDS-SF-Geriatric Depression Scale Short Form (range; 0 = no indication of depression, 15 = high possibility of depression).

^d^ STAI-Spielberger State or Trait Anxiety Inventory (range; 20 = indicates increased anxiety, 80 = indicates least amount of anxiety).

Summaries of the temperature at which each of the pain percepts were reported by the participants and their perceptions of the unpleasantness of those pain percepts are shown in Table 2. As expected, statistically significant main effects of increasing pain percept levels were observed on both the stimulus temperatures and pain unpleasantness ratings, confirming the efficacy of the evoked pain protocol used in this study (*p* < 0.001). There were statistically significant main effects of AD status on the perception of pain temperatures (*p* < 0.001) and on reports of pain unpleasantness (*p* = 0.039). Participants with AD reached a higher temperature before reaching the pain percepts compared to controls (i.e., less sensitive to sensory pain). Furthermore, when pain was detected, participants with AD reported that pain to be more unpleasant than controls. The interaction effect of AD status and pain level on temperature and affect were not statistically significant (temperature: *p* = 0.622; affect: *p* = 0.168). The effect size for the influence of AD on percept temperatures were strongest at the warmth (Cohen's *d* = 0.91) and mild pain (*d* = 0.90) levels. For pain unpleasantness ratings, the strongest effect of AD status was at the level of mild pain (Cohen's *d* = 0.60, see Table 2).

**Table 2:** Psychophysics of temperature thresholds necessary to produce percepts of warmth, mild pain, and moderate pain, as well as unpleasantness ratings (pain affect) at each percept level by study group.

	Total (*N* = 36)	Control (*N* = 18)	AD (*N* = 18)	Cohen's *d*	*p*-value for overall group effect
	Median [IQR] Min, Max	Median [IQR] Min, Max	Median [IQR] Min, Max		
Temperature °C					<.001
Warmth	33.0 [32.0, 34.0] 31, 38	32.0 [32.0, 33.0] 31, 35	34.0 [33.0, 35.0] 32, 38	0.91	
Mild pain	36.0 [35.0, 39.8] 33, 46	35.0 [34.0, 37.0] 33, 46	39.0 [35.8, 40.3] 34, 43	0.90	
Moderate pain	41.5 [38.0, 43.8] 34, 48	39.5 [38.0, 42.0] 34, 48	43.0 [39.0, 44.3] 36, 48	0.71	
Affect^a^					0.039
Warmth	0.0 [0.0, 0.0] 0, 4	0.0 [0.0, 0.3] 0, 3	0.0 [0.0, 0.0] 0, 4	0.01	
Mild pain	3.5 [0.2, 5.0] 0, 11	1.5 [0.0, 4.0] 0, 5	4.0 [0.7, 5.0] 0, 11	0.60	
Moderate pain	6.0 [4.2, 8.0] 0, 14	5.0 [3.7, 6.3] 0, 11	7.0 [4.2, 10.3] 1, 14	0.35	

^a^ 0–20 unpleasantness scale (0 = neutral, 20 = very intolerable) (49).

Confirming the sensory inducement paradigm, statistically significant main effects of increasing threshold levels were observed on both temperature and unpleasantness (*p *< 0.001). Neither of the interaction effects of AD status and threshold level were statistically significant (temperature: *p* = 0.622; unpleasantness: *p* = 0.168).Values were square-root transformed to meet normal distribution assumptions of linear models.

**Figure 1:**
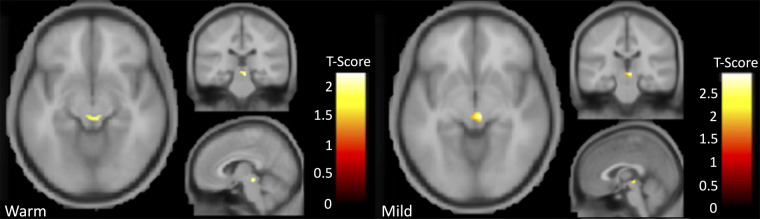
Results indicating greater activation in AD participants relative to controls in the PAG for the contrast comparison of warmth greater than pre-stimulus (left) and mild pain greater than baseline (right).

BOLD responses in the PAG during the heat-induced pain paradigm were greater in participants with AD compared to controls during warmth and mild pain (Figure 1 and Table 3 for comparison data) but there was no significant group difference during moderate pain. There were no significant differences in BOLD responses in the insula, thalamus, ACC, or S2 between participants with AD and controls during warmth, mild, or moderate pain as no voxels surpassed the FWEc threshold (*p* > 0.05).

**Table 3:** Group differences in PAG ROI BOLD responses during warmth and mild pain.

Comparison of all activations results above the threshold in the PAG	Extent/voxel threshold for cluster correction^a^	Number of voxels in cluster	Peak T (T-score)	Peak coordinates (x, y, z)	*p*-value
Control < AD during warmth (Figure 1, left)	K = 10	K_E_ = 28	2.22	6, −30, −12	0.016
Control < AD during mild pain (Figure 1, right)	K = 10	K_E_ = 33	2.96	6, −24, −6	0.003

^a^ measured in units of contiguous voxels.

**Figure 2:**
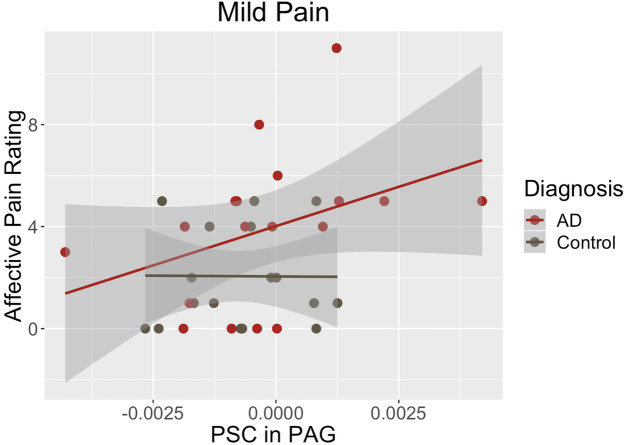
During mild pain, the correlation of PAG percent signal change with affective ratings was statistically significant in the complete sample (*r_s _*= 0.34, *p* = 0.042). That effect was driven by a strong correlation within the Alzheimer’s group (*r_s _*= 0.54, *p* = 0.020).

Finally, we assessed the relationships of PAG BOLD activations with each psychophysical variable. Those correlations for the entire sample and within each study group are shown in Table 4. None of the correlations between PAG percent signal change and stimulus temperature needed to elicit the pain percepts (i.e., sensory pain) were statistically significant. The strongest correlation was observed for the entire group during the initial warmth percept (*r_s _*= 0.32, *p* = 0.056). The strongest correlation between PAG percent signal change and affective pain ratings was observed during the mild pain percept and was statistically significant (*r_s _*= 0.34, *p* = 0.042). Most of that effect, however, was from an even stronger correlation within the AD group (*r_s _*= 0.54, *p* = 0.020) (Table 4, Figure 2). The sample was too small to detect any statistically significant differences between the group in the sizes or directions of the within-group correlations (*p* > 0.05, Table 4).

**Table 4:** Correlations of the PAG percent signal change with the stimulus temperature needed to elicit each pain percept and affective ratings at each percept for the entire sample and within each group.

Percept	PAG % Signal Change			
	All (*N* = 36)	Control (*N* = 18)	AD (*N* = 18)	*p*-value^a^
Warm				
Temperate	0.32 (0.056)^a^	0.20 (0.424)	0.22 (0.387)	0.477
Affect	−0.27 (0.114)	−0.33 (0.182)	−0.20 (0.416)	0.351
Mild Pain				
Temperate	0.05 (0.758)	−0.04 (0.882)	−0.07 (0.793)	0.467
Affect	0.34 (0.042)	0.10 (0.702)	0.54 (0.020)	0.084
Moderate Pain				
Temperate	0.05 (0.755)	0.12 (0.631)	−0.12 (0.636)	0.254
Affect	−0.06 (0.720)	0.12 (0.628)	−0.15 (0.557)	0.228

^a^ *p*-value for z-test of differences between independent correlations.

## Discussion

Pain is often underrecognized and undertreated in people with AD. There is growing evidence that people with AD may be at greater risk for mismanaged pain due to differences in pain reporting and pain processing (1, 2, 5, 49, 86). We sought to examine PAG response to pain in AD, given its role in pain modulation (23, 30, 31, 71). We found that PAG BOLD activations were significantly greater in people with AD than in cognitively intact controls during mild pain stimuli, and that these PAG activations were significantly associated (positively) with affective pain ratings (pain unpleasantness) only in those with AD. This work underscores the importance of understanding biological differences in pain processing when evaluating differences in pain reporting in AD. Findings furthermore support the use of multi-dimensional pain assessment practices (87) and tools (88) since most clinicians and clinical systems use a simple 0–10 scale assessing only the sensory rather than affective component of pain (89, 90). This work suggests alternative assessment strategies to better capture altered pain responses in AD, reducing the potential for poorly managed pain and unnecessary suffering in this population.

### Psychophysical findings

The protocol used in this study was confirmed to effectively induce pain, with increasing stimulus temperatures and unpleasantness ratings with more intense pain percept targets. Although the interaction effect of AD status and pain percept level was not statistically significant, participants with AD reported reaching all percepts at higher temperatures than controls (i.e., AD participants could be less pain sensitive than controls or require additional time to recognize pain), with effect sizes that may indicate a meaningful difference. The current study simply did not have sufficient power to detect these differences. These psychophysical findings demonstrate that participants with AD may need more intense experimental stimuli to report perceptually-matched pain percepts, a finding consistent with previous findings from the parent psychophysics study (49) and in general.[as reviewed in (5)] However, the experience of pain in people with AD may be more unpleasant compared to controls, a finding in this study supported by significant linear associations across all percepts. Increased pain unpleasantness is also consistent with several previous findings (1, 5, 6, 44, 47, 51–54). While comparisons at the individual percept levels were not significant, participants with AD rated mild and moderate pain affect as more unpleasant than controls, again with effect sizes that may indicate a meaningful difference. These findings suggest that pain may be perceived as more unpleasant in the person with AD compared to healthy older adults and that people with AD may continue to suffer from pain longer before recognizing and reporting this pain, potentially delaying treatment.

One potential mechanism for higher percept thresholds in AD could be related to PAG function. Activation of the PAG elicits descending inhibition of nociceptive information through the rostral ventromedial medulla (RVM), which acts as a relay station to the spinal cord dorsal horn (31, 32, 36, 91). The PAG-RVM pathway is crucial for pain modulation (28, 60, 92), and influences withdrawal reflexes that help guard the body from injury (93). Inactivation of this pathway can alter pain withdrawal thresholds (93), and damage to this area of the midbrain found in AD (19–22) may mean that withdrawal reflexes to pain are altered. This may be a contributing factor as to why participants with AD needed greater stimuli, or more time, to process or verbally report pain. Greater pain unpleasantness is discussed in relation to the PAG below.

### fMRI response in the PAG

Neuroimaging findings from this study demonstrate that PAG activation was significantly greater during an experimental evoked heat pain protocol in participants with AD compared to controls during the experience of warmth and mild pain (Figure 1 and Table 3). During mild pain, while the difference was not statistically significant, a stronger and statistically significant positive correlation of percent signal change in the PAG with affective/unpleasantness ratings was observed for participants in the AD group but not for controls (Table 4, Figure 2).

These findings indicate that input to the PAG is at least partially preserved in AD. Some ongoing modulation of pain *via* the PAG is suggested by the fact that there was not an absence of activation in the PAG in people with AD and because unpleasantness ratings correlated with percent signal change in the PAG in the AD group. It is possible that the fact that these differences as a function of AD status only occurred at mild pain but not moderate pain intensity may indicate a lowered threshold for activation of the PAG in those with AD, with both groups displaying PAG activation at higher stimulus intensities. However, the pattern of findings regarding altered PAG activity in people with AD may be complex to interpret. For example, increased sensory pain intensity has been found to be associated in cognitively intact individuals with increased PAG activation (60, 61), presumably reflecting the afferent arm of the descending pain inhibitory system (i.e., nociceptive activity that leads to PAG activation without the expected pain reduction occurring with efferent PAG activity). To the extent that the current findings in those with AD of apparently lower sensitivity to sensory pain (i.e., higher percept temperatures) may be due to delayed reporting of percepts, as might be suggested by findings regarding withdrawal from pain noted above. The greater stimulus temperatures experienced by the AD group might be expected to lead to greater activation of the afferent PAG pathway leading to positive associations. Given the brevity of the pain stimuli in this study, it is conceivable that impact of the efferent side of PAG activation (opioid release) may not have had time to inhibit the experience of the affective pain component (i.e., resulting in elevated unpleasantness ratings in the AD group). Alternatively, it is possible that opioid system function may be altered in AD (94) with a decrease in receptor binding sites (95) and abnormal variations in receptor binding (94). It is thus possible that although afferent pathways through which pain stimuli trigger PAG activity are preserved in AD, the resulting endogenous opioid release, or analgesic effects of those released opioids, may be impaired by other AD-related changes. If the endogenous opioid system has significant dysfunction in AD, this could mean that people with AD have a reduced ability to modulate pain in the absence of pharmacological intervention in the clinical context.

### Limitations and future research

There are several factors that could limit interpretation of these initial study findings. The study design included a perceptually-matched pain protocol and participants with AD required higher temperatures before they reported pain. While this gives us information about their subjective experience of pain, this could be a reason why there is greater activation in the PAG, as the higher temperature could potentially cause greater induction of pain modulatory systems. Replication of our findings with a fixed temperature paradigm would aid in interpretation of these results. In fact, a fixed heat pain paradigm would also reduce cognitive burden on participants with AD, lessening the adverse impact of decision making and reaction time on the psychophysical responses. Fixed temperatures would also allow for inclusion of more severe AD in future psychophysical research, which is greatly needed.

The current study did not examine functional connectivity, which limits our ability to determine how the PAG is related to other pain regions. Dysfunction from pathological changes in the PAG during the disease process of AD, as well as resultant altered connectivity with the wider pain network, could impact pain processing in AD. Likewise, there is the potential that central sensitization could differ in AD but current studies have not addressed this source of pain variability in AD, indicating another area for future work. Additionally, because this study could not include non-communicative participants, these results may not be generalizable to people with severe AD. However, given our results, our work certainly suggests that it is likely that people with severe AD still experience and suffer from pain.

Future research needs to include larger sample sizes to enable detection of potentially meaningful differences between the AD and non-AD control groups and to examine potential sex differences in PAG function during pain, given known differences in pain perception by sex (63, 96–98). In the current work, potential confounds related to sex were addressed by sex matching of the AD and control samples. In future studies using larger samples, with inclusion of more severe stages of AD, it will be important to understand the evolution of PAG function across AD progression as pathological studies suggest the PAG may be targeted by early AD pathology (4). Additionally, although limited, there is evidence of opioid receptor dysfunction in AD (94) but how this impacts pain processing and the experience of pain has not been studied. Given the role of the PAG in endogenous opioid system function (30, 32–34, 36) and exogenous opioid responsiveness (36, 38–40), it will be critical to integrate knowledge of endogenous opioid function and the PAG in the observed aberrant pain processing in AD. Because the PAG plays a substantial role in the effects of exogenous opioid analgesics (36, 38–40), PAG functional alterations could impact the efficacy of opioid analgesics in AD, with potential implications for opioid prescribing in AD patients with acute or chronic pain. In our study participants with chronic pain were screened out, however, future work should examine responses in people living with AD and chronic pain.

## Conclusion

Our current findings suggest that the PAG may play a role in altered pain responses in people living with AD. Because greater PAG responses were significantly associated with higher reports of pain unpleasantness only in people with AD, our data suggests people with AD may be experiencing pain differently than cognitively intact individuals due in part to altered brain function. Endogenous processing and top-down modulation of pain in those with AD may be disrupted. Our psychophysical findings suggest that people with AD could be undertreated for pain, and thus, have a greater risk of suffering from pain compared to cognitively intact older adults. Given communication difficulties sometimes associated with AD (51, 54), clinicians and caregivers should be mindful that people with AD likely feel pain but may have difficulty initiating communication of their pain or accurately conveying their pain (87, 99, 100).

## Data Availability

The original contributions presented in the study are included in the article/supplementary materials, further inquiries can be directed to the corresponding author/s.
